# Evaluation of the Mechanical Properties of Three Resin-Modified Glass-Ionomer Materials

**DOI:** 10.1155/2022/4690656

**Published:** 2022-08-02

**Authors:** Heleine Maria Chagas Rêgo, Sheila Butler, Maria Jacinta Coelho Santos

**Affiliations:** Division of Restorative Dentistry, Schulich School of Medicine and Dentistry, Western University, Ontario, Canada

## Abstract

This study is aimed at evaluating the flexural strength (FS), fracture toughness (FT), and diametral tensile strength (DTS) of three resin-modified glass-ionomer cements (RMGICs): Ketac Nano, Riva Light Cure, and Fuji II LC. One hundred twenty specimens were prepared from the RMGIC materials (*n* = 10). The cements were mixed and inserted into different mould sizes according to the test performed: FS: rectangular Teflon mould (32 mm × 3.15 mm × 2 mm); FT: notchless triangular prism (NTP) Teflon mould (6 mm × 6 mm × 6 mm × 12 mm); and DTS: ring road stainless steel mould (6 mm × 3 mm). Specimens were light cured for 20 seconds on each surface and stored in distilled water at 37°C ± 2°C for seven days prior to tests. To evaluate the influence of storage in the mechanical properties of the RMGIs, specimens tested for DTS were stored in distilled water at 37°C ± 2°C for 32 days prior to test. Data were analyzed by ANOVA and Tukey's test (*α* = 0.05). Fuji II LC presented significantly higher values for all tests employed when compared to Ketac Nano and Riva LC RMGIs. There was no significant difference on DTS before and after the 32-day storage for each material. Fuji II LC presented superior mechanical properties when compared to Ketac Nano, and Riva LC storage showed no influence on the mechanical properties of the RMGI materials tested.

## 1. Introduction

Glass-ionomer cements (GICs) were introduced in dentistry by Wilson and Kent in 1970s [1–3]. An acid-base reaction between a calcium fluoroaluminosilicate glass powder and an aqueous solution of polyacrylic acid constitutes the main feature of GIC materials. As a result, ionically cross-linked polymer chains with multivalent counter ions leached from the glass are formed, ending in a self-hardening material [4–8].

The popularity of ionomer-based materials is related to their relevant features, such as chemical adhesion to dental structures, biological sealing of dentin, biocompatibility, coefficient of thermal expansion similar to the dental structure, and anticariogenic properties due to their fluoride release [9–17]. Despite their advantages, undesirable characteristics have been reported, such as inferior mechanical properties, limited esthetic, and difficulty in handling when compared to resin composite materials [7, 12, 15]. Since the introduction of GICs, several modifications in the material's composition were performed to minimize undesirable features that could limit their clinical use. The incorporation of hydrophilic resin monomers into an aqueous solution of polyacrylic acid led to the development of a more resistant, resin-modified glass-ionomer material (RMGI). RMGIs exhibit improved mechanical properties, while keeping the desirable characteristics of the conventional GICs [3, 7, 16–21]. Regarding their elastic behavior, GICs and RMGIs are polymer-based composites and may potentially exhibit viscoelastic behavior. Typically, the viscoelastic properties of these materials are calculated using creep evaluations [22, 23]. The elastic behavior of these materials varies significantly from brand to brand [24].

RMGI materials presenting silane-treated silica nanofillers and nano-sized zirconia/silica clusters, with a highly packed filler composition, were developed [15, 16, 25]. Additionally, easy-handling materials, supplied in capsules, are available, allowing for an ideal powder/liquid ratio and automatic mixing, facilitating the manipulation, and optimizing the materials' properties [5, 26–30]. The superior mechanical properties, easy handling, and improved esthetic results presented by RMGIs have enabled their use in the permanent dentition. Significantly higher retention rates have been reported for their use in noncarious cervical lesion restorations when compared to resin composite restorations [31].

The improved mechanical properties achieved by RMGI materials have been reported by long-term clinical studies. Donly et al. and Espelid et al. [32, 33] evaluated the clinical performance of RMGI class II restorations in primary teeth and observed a similar performance when compared to amalgam and a better performance when compared to silver cermet, respectively, for this material over 36 months. Dulgergil et al. [34] studied the RMGI for ART and noted superior clinical performance over six months, compared to GIC materials. Fagundes et al. [35] carried RMGI restorations in noncarious cervical lesions and observed 95.8% of cumulative survival rate of retention of this material over seven years.

Due to the importance of evaluating the mechanical properties of recent RMGIs available, this study is aimed at evaluating the flexural strength (FS), fracture toughness (FT), diametral tensile strength (DTS), and the effect of storage in three resin-modified glass-ionomers (RMGIs) available in capsules. The null hypotheses are: (1) there would be no significant difference in FS, FT, and DTS among the three materials tested; (2) there would be no significant differences in DTS of the RMGI materials tested after storage.

## 2. Materials and Methods

### 2.1. Specimen Preparation

A total of 120 specimens were fabricated using the following RMGIs: Ketac Nano (3M ESPE, St. Paul, MN, USA), Riva Light Cure (SDI, Bayswater, VIC, Australia), and Fuji II LC Capsule (GC America, Alsip, IL, USA), according to the manufacturer's instructions (Table 1). The specimens' preparation was followed by ISO 9917-2: 2017 [36].

**Figure 1:**
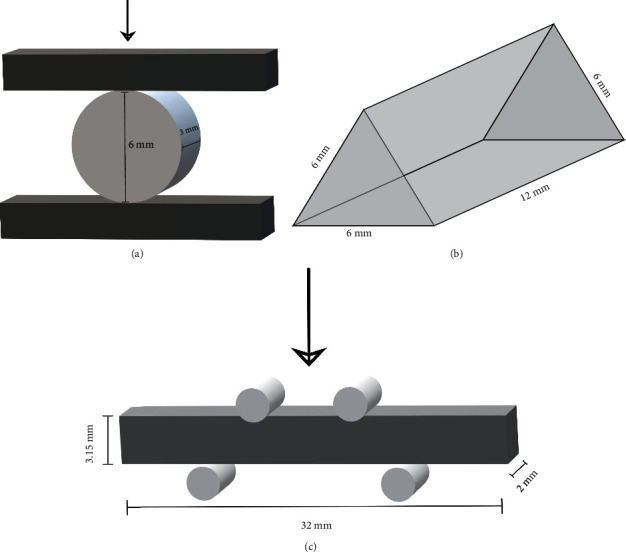
Illustration of the specimens prepared for the tests: (a) diametral tensile strength; (b) fracture toughness; (c) flexural strength.

Specimens were fabricated in moulds with different dimensions according to the test performed: flexural strength test (FS) (*n* = 10): rectangular Teflon mould (32 mm × 3.15 mm × 2 mm); fracture toughness test (FT) (*n* = 10): notchless triangular prism (NTP) Teflon mould (6 mm × 6 mm × 6 mm × 12 mm); and diametral tensile strength test (DTS) (*n* = 10) prepared using a ring road stainless steel mould (6 mm × 3 mm) (Figure 1).

The RMGI materials that required mixing were manipulated in an amalgamator (Ultramat 2, SDI, Bayswater, VIC, Australia) for 10 seconds. After mixing, the capsule was loaded into a gun applicator, and the material was inserted into the mould. A clear glass cover slip (microslides, Gold Seal) was placed on top of the material, and a gentle pressure was applied to extrude material excess and to obtain a flat and smooth surface. The RMGI material was light-cured for 20 seconds using a LED curing-light unit (Bluephase Style, Ivoclar Vivadent, Mississauga, ON, Canada, light intensity of 1330 mW/cm^2^). Additional curing (20 seconds) was performed on each side of the specimen after they were removed from the mould to assure complete cure throughout the material. All specimens were inspected for flaws using a back light, and the defective ones (showing cracks, porosity, or lack of material) were discarded. Two specimens of Riva LC, none specimens of Fuji II LC, and two specimens of Ketac Nano for FS test were discarded. Two specimens of Riva LC, two specimens of Fuji II LC, and one specimen of Ketac Nano for FT were discarded. Three specimens of Riva LC, two specimens of Fuji II LC, and three specimens of Ketac Nano for DTS test were discarded. After fabrication, specimens were stored in distilled water at 37°C ± 2°C for seven days prior to test. To evaluate the influence of a 32-day storage, specimens (*n* = 10) from each material were kept in distilled water at 37°C ± 2°C under constant agitation. The water was changed weekly until the DTS test was performed as an equilibrium of ions transfer between the sample, and unchanged storage solution would be established in the solution. Additionally, changing the storage solution may also have accelerated the aging process of the samples [7, 37–41].

The mechanical tests were performed using a universal testing machine (Instron—Model 3345, Norwood, MA, USA).

### 2.2. Flexural Strength Testing

The test was performed in four-point bending, with a span of 30 mm between supports, at a crosshead speed of five mm/min; the test is in accordance with the ISO 5833 specification [42]. The FS was calculated following the formula:

(1) $$\begin{array}{c} \sigma =\frac{3PL}{2{bd}^{2}} \end{array}$$

where *P* is the maximum load, *L* is the distance between the two supports, *b* is the breadth of the specimen, *d* is the depth of the specimen, and *σ* is the FS value expressed in MPa (N/mm^2^).

### 2.3. Fracture Toughness Testing

The samples were scorched at the location of tensile forces in order to create a defect. Force was applied until failure of the FT (*K*_IC_) as proposed by Barker [43] and adopted by ASTM standard El304. The FT was calculated using the formula:

(2) $$\begin{array}{c} K_{\text{IC}}=\frac{P_{\text{MAX}}}{\;D\surd W}*Y^{*}\min \end{array}$$

where *P*_MAX_ is the load at fracture, *D* is the specimen diameter, *W* is the specimen length, *Y*^∗^_min_ is the minimum of the dimensionless stress intensity factor coefficient (=28), and *K*_IC_ is the FT value expressed in MPa√m.

### 2.4. Diametral Tensile Strength Testing

Specimen dimensions were measured before testing. Test was performed using a rounded rectangular rod testing device at a crosshead speed of 0.5 mm/min. The DTS was calculated following the formula [20]:

(3) $$\begin{array}{c} \text{DTS}=\frac{2L}{pdh} \end{array}$$

where *L* is the load of fracture, *p* = 3.14, *d* is the diameter of the samples, and *h* is the height of the samples. DTS values (kgf/cm^2^) were converted into MPa as follows: DTS (MPa) = DTS (kgf/cm^2^) × 0.09807.

### 2.5. Statistical Analysis

Data were analyzed using one-way ANOVA and Tukey post hoc tests (*α* = 0.05). A two-way ANOVA was also performed to evaluate the differences between the materials tested (DTS) after storage. The raw data is available as a Supplementary Material.

## 3. Results

Mean values and standard deviations of FS, FT, and DTS obtained from the RMGI materials are presented in Tables 2 and 3.

Fuji II LC presented significantly higher FS, FT, and DTS compared to Ketac Nano and Riva Light Cure. There were no statistically significant differences between Ketac Nano and Riva Light Cure for all tests performed (*p* > 0.05). In addition, no significant differences were observed before and after 32-day storage for all RMGI materials tested (*p* > 0.05).

## 4. Discussion

For this investigation, three tests were selected to evaluate FS, FT, and DTS of different RMGICs. According to the results obtained in the present study, the Fuji II LC RMGI material presented significant higher values of FS, FT, and DTS than the other RMGI materials tested (Ketac Nano and Riva Light Cure). Based on these data, the null hypothesis, which stated no significant differences in the mechanical properties for the different types of RMGIs tested, was rejected. These results are in agreement with other studies that have verified superior performance of Fuji II LC [3, 4, 21, 25, 44, 45].

Compared to conventional glass ionomer cements (GI), RMGI exhibit increased hardness, fracture toughness, flexural strength, diametral tensile strength, and wear resistance [4, 46–49]. The presence of resin components contributes to the superior mechanical properties, a shortened setting time, decreased early moisture sensitivity, extended working time, increased translucency, and superior esthetic results [17, 48, 50]. Additionally, RMGI presents increased ability to deform plastically under load, resulting in increased fracture toughness [4, 46].

RMGIs have been indicated for the treatment of atraumatic restorative treatment (ART) in permanent and primary teeth [34]; restoration of classes I, II, III, and V in primary teeth [33, 47, 48, 51, 52]; and small classes I, III, and V in permanent teeth [48] and have been indicated to be used in sandwich combination with composite resin materials in class II preparations located below the cement-enamel junction (CEJ) [27]. A previous study reported that the combination of resin composite and glass ionomer liner materials may reduce some of the residual stresses during polymerization shrinkage and loading [53]. Additionally, RMGI has shown the highest retention rate in noncarious cervical lesions (NCCL) compared to resin composite. The restoration of NCCLs is often considered a challenging procedure since partial or complete obliteration of the dentinal tubules with sclerotic casts (crystallites) and a hypermineralized layer is often present on those lesions as a natural defence to insult. Those barriers prevent primer diffusion and resin infiltration resulting in reduced bond strength to dentin; for that reason, GI and RMGI materials have become the most common materials used to restore NCCLs due to the chemical adhesion achieved between calcium in hydroxyapatite and carboxyl groups from the polyalkenoic acid (PAA) [31, 35].

In the present study, three RMGIs delivered in capsules were selected to better standardize the material powder/liquid proportion and allow a more accurate interpretation of the results [46, 49, 54], as previous studies have shown that variations in the powder/liquid ratio may negatively interfere in the mechanical properties [5, 55]. Moreover, the spatulation of powder and liquid in hand mixed materials leads to increase air bubble incorporation and micropores that can compromise the mechanical properties of the restorative materials. In contrast, RMGI delivered in automix capsules allows for an ideal powder/liquid ratio and minimum incorporation of air bubbles via the mixing process, while facilitates handling [5, 8, 20, 27, 56].

In a previous study, Fuji II LC and Ketac Nano presented no significant difference in flexural strength [57]. This is not in agreement with the present study, and it may be attributed to the fact that the samples were tested in a three-point flexure, while in the present investigation, a four-point flexure was used. However, after 84 days in storage, Fuji II LC exhibited a 50% decrease in the FS values compared to a 61% decrease for Ketac Nano. Despite the incorporation of zirconia to Ketac Nano composition, the mechanical property values of Ketac Nano were lower when compared to Fuji II LC in the present investigation. Ketac Nano RMGI combines the features of an acid-reactive fluoroaluminosilicate glass and nonreactive nanofillers, resulting in a highly packed filler composition (~69% weight), with optimized esthetic and polishability [25, 45, 48]. The superior polishability and improved resistance to abrasion have been verified in clinical studies [44, 47, 48]. A literature review reported that Ketac Nano did not present superior mechanical properties over microfilled RMGICs when tested for flexural strength and tensile strength. In theory, the addition of zirconia nanoparticles to the GIC composition improves the mechanical properties and reduces porosities; however, studies have shown that it depends on the amount of particles added, which vary in different commercial materials [58–60].

A correlation between volume, filler size, and shape on fracture toughness load values has been observed for resin-based materials [54, 61–63]. The higher the filler size and/or volume of fillers are/is, the higher the FT values are, with a greater initial value of the stress-intensity factor for crack, regardless of the degree of conversion [50, 61–63]. The same correlation can also be applied for the RMGI materials tested in this study. The Fuji II LC RMGI presents predominantly large particles (25 *μ*m) [54] while Ketac Nano incorporates nanoparticles (5-25 nm), nanoclusters (1-1.6 microns), and fluoroaluminosilicate glass (1 micron). The highest FT values obtained for Fuji II LC can be attributed to the presence of larger particle size in this material.

Furthermore, in a previous investigation, a better performance of Fuji II LC on FS and FT was observed after one week storage when compared to Ketac Nano and Riva Light Cure [8]. In the present evaluation, Riva Light Cure exhibited similar mechanical property values compared to Ketac Nano and significant lower values compared to Fuji II LC. The similar mechanical property values between Ketac Nano and Riva Light Cure can be attributed to the similar filler content; Ketac Nano exhibits 69% by weight (according to the manufacturer's instructions), and Riva Light Cure presents 72.96 wt% filler content [64]. In addition, the highest values of FS and DTS presented by Fuji II LC have been previously attributed to a better integrated interface between the glass particle and polymer matrix [4] and present 76.2 wt% [57]. The standard deviations of the samples submitted to the DTS test were similar to previous studies [60, 65, 66].

In the present study, DTS was performed before and after storage of the specimens [4, 10, 14, 20]. DTS was initially investigated after a seven-day storage and after a 32-day storage period. One disadvantage of resin-modified glass ionomer is the hydrophilic nature of poly-hydroxyethyl methacrylate, which results in increased water absorption and subsequent plasticity and hygroscopic expansion. The plasticizing action of the water can affect the materials by reducing their mechanical properties. The 32-day storage is aimed at verifying the influence of the water absorption on the DTS [67]. No significant difference was observed between the two storage periods; thus, the second null hypothesis was accepted. These findings are in agreement with previous evaluations [3] that observed no significant differences on the mechanical properties (compressive strength, compressive modulus, and diametral tensile strength) of Fuji II LC materials submitted to different storage periods (24 hours, one week, four weeks, 12 weeks, 24 weeks, and 52 weeks). A previous study [68] also reported no changes in compressive strength and diametral tensile strength of RMGICs tested after different storage periods. Zankuli et al. [69] observed no significant differences in compressive strength between Fuji II LC before and after cycling loading, concluding that this restorative material could survive one year in service without a decrease in these mechanical properties. Additionally, a previous study observed an increase in the compressive strength of Fuji II LC at a *P/L* ratio of 1 : 3 after 28 days of storage in water. The authors explained the importance of water sorption in the aging process of glass ionomer materials and its influence on their mechanical strength since storage times are related to beneficial factors that increase strength, such as hydration of metal-carboxylate links and maturation of the polysalt matrix, and other detrimental factors, such as polymer matrix hydrolysis. Thus, the combination of those factors may explain the results generated in the present study and previous studies [70].

Moberg et al. [8] observed no difference in FT of Fuji II LC, Ketac Nano, and Riva Light Cure after one-week and one-month storage. When evaluating FS, no difference was observed after one-week and one-month storage for Fuji II LC and Riva Light Cure. On the other hand, Ketac Nano showed reduced flexural strength after one-month storage. The RMGIs' stability in water verified in the present study may be related to the immediate hardening after the light-curing reaction [4, 46–48, 51, 56], as well as their presentation in automix capsules, which allows for an ideal powder-liquid proportion, eliminating the possibility of compromising the mechanical properties due to undesirable proportion and mixing [5, 20, 28–30].

This in vitro study presented some limitations. A longer storage time and the use of cycling load and fatigue stresses could have contributed to creating a more challenging environment before testing the specimens. This study followed the methodology presented in other investigations to allow comparisons with previous studies. Although in vitro studies can generate important information about materials' properties, clinical trials will reveal their clinical performance and longevity.

## 5. Conclusion

Within the limitations of this in vitro study, it was possible to conclude that the values of flexural strength, fracture toughness, and diametral tensile strength were superior for the Fuji II RMGI when compared to Ketac Nano and Riva Light Cure. The 32-day storage did not affect the mechanical properties of the RMGIs tested.

## Figures and Tables

**Table 1:** Composition of the RMGI materials used in this study.

Material	Composition	Batch number	Manufacturer
Ketac Nano	De-ionized water, 2-hydroxyethyl methacrylate, Vitrebond copolymer (a methacrylate modified polyalkenoic acid) Silica/zirconia with nanofillers (5-25 nm) and nanoclusters (1-1.6 micron) Radiopaque particles of fluoroaluminosilicate glass (1 micron) Inorganic filler with 69% by weight or 56% by volume	N511981	3M ESPE, St. Paul, MN, USA
Riva Light Cure	Polyacrylic acid (15-25%) Tartaric acid (1-5%) 2-Hydroxyethyl methacrylate (20-25%) Dimethacrylate cross-linker (10-25%) Acidic monomer (10-20%) Fluoroaluminosilicate glass powder (95-100%)	J130422EG	SDI, Bayswater, VIC, Australia
Fuji II LC capsule	Polyacrylic acid (20-25%) 2-Hydroxyethyl methacrylate (30-35%) Proprietary ingredient (5-15%) 2,2,4-Trimethyl hexamethylene decarbonate (1-5%) Alumino-fluorosilicate glass (95-100%)	1401251	GC America, Alsip, IL, USA

**Table 2:** Flexural strength and fracture toughness in MPa, mean, and standard deviation.

Material	Flexural strength	Fracture toughness
	Mean (±sd)	
Ketac Nano	14.04 (1.42)^B^	0.15 (0.03)^B^
Riva Light Cure	15.67 (1.96)^B^	0.20 (0.03)^B^
Fuji II LC Capsule	37.60 (5.33)^A^	0.27 (0.08)^A^

Mean values followed by different small letters in the column differ statistically among themselves for the Tukey test at the level of 5%.

**Table 3:** Diametral tensile strength in MPa, mean, and standard deviation.

Material	7-day storage	32-day storage
	Mean (±sd)	
Ketac Nano	10.78 (2.19)^B^	13.74 (4.80)^B^
Riva Light Cure	13.30 (2.54)^B^	12.92 (3.27)^B^
Fuji II LC Capsule	20.93 (4.04)^A^	20.52 (3.16)^A^

Mean values followed by different small letters differ statistically among themselves for the Tukey test at the level of 5%.

## Data Availability

The data is available upon request.

## References

[1] Wilson A. D., Kent B. E. (1971). The glass-ionomer cement, a new translucent dental filling material. *Journal of Applied Chemistry and Biotechnology*.

[2] Wilson A. D., Kent B. E. (1972). A new translucent cement for dentistry. The glass ionomer cement. *British Dental Journal*.

[3] Mitra S. B., Kedrowski B. L. (1994). Long-term mechanical properties of glass ionomers. *Dental Materials*.

[4] Xie D., Brantley W. A., Culbertson B. M., Wang G. (2000). Mechanical properties and microstructures of glass-ionomer cements. *Dental Materials*.

[5] Fleming G. J., Farooq A. A., Barralet J. E. (2003). Influence of powder/liquid mixing ratio on the performance of a restorative glass-ionomer dental cement. *Biomaterials*.

[6] Kim D. A., Abo-Mosallam H. A., Lee H. Y., Kim G. R., Kim H. W., Lee H. H. (2014). Development of a novel aluminum-free glass ionomer cement based on magnesium/strontium-silicate glasses. *Materials Science & Engineering*.

[7] Shiozawa M., Takahashi H., Iwasaki N. (2014). Fluoride release and mechanical properties after 1-year water storage of recent restorative glass ionomer cements. *Clinical Oral Investigations*.

[8] Moberg M., Brewster J., Nicholson J. W., Roberts H. (2019). Physical property investigation of contemporary glass ionomer and resin-modified glass ionomer restorative materials. *Clinical Oral Investigations*.

[9] Yli-Urpo H., Lassila L. V., Närhi T., Vallittu P. K. (2005). Compressive strength and surface characterization of glass ionomer cements modified by particles of bioactive glass. *Dental Materials*.

[10] Yamazaki T., Schricker S. R., Brantley W. A., Culbertson B. M., Johnston W. (2006). Viscoelastic behavior and fracture toughness of six glass-ionomer cements. *The Journal of Prosthetic Dentistry*.

[11] Bonifácio C. C., Kleverlaan C. J., Raggio D. P., Werner A., de Carvalho R. C., van Amerongen W. E. (2009). Physical-mechanical properties of glass ionomer cements indicated for atraumatic restorative treatment. *Australian Dental Journal*.

[12] Zhao J., Weng Y., Xie D. (2009). In vitro wear and fracture toughness of an experimental light-cured glass-ionomer cement. *Dental Materials*.

[13] Fabián Molina G., Cabral R. J., Mazzola I., Brain Lascano L., Frencken J. E. (2013). Biaxial flexural strength of high-viscosity glass-ionomer cements heat-cured with an LED lamp during setting. *BioMed Research International*.

[14] Howard L., Weng Y., Xie D. (2014). Preparation and evaluation of a novel star-shaped polyacid-constructed dental glass-ionomer system. *Dental Materials*.

[15] Abo-Hamar S. E., El-Desouky S. S., Abu Hamila N. A. (2015). Two-year clinical performance in primary teeth of nano-filled versus conventional resin-modified glass-ionomer restorations. *Quintessence International*.

[16] Najeeb S., Khurshid Z., Zafar M. S. (2016). Modifications in glass ionomer cements: nano-sized fillers and bioactive nanoceramics. *International Journal of Molecular Sciences*.

[17] Lagarde M., Francois P., Goff S. L., Attal J. P., Dursun E. (2018). Structural and long-term mechanical properties from a resin-modified glass ionomer cement after various delays of light-activation. *Dental Materials Journal*.

[18] Walls A. W. (1986). Glass polyalkenoate (glass-ionomer) cements: a review. *Journal of Dentistry*.

[19] Wilson A. D. (1989). Developments in glass-ionomer cements. *International Journal of Prosthodontics*.

[20] Molina G. F., Cabral R. J., Mazzola I., Lascano L. B., Frencken J. E. (2013). Mechanical performance of encapsulated restorative glass-ionomer cements for use with atraumatic restorative treatment (ART). *Journal of Applied Oral Science*.

[21] Bilić-Prcić M., Rajić V. B., Ivanišević A., Pilipović A., Gurgan S., Miletić I. (2020). Mechanical properties of glass ionomer cements after incorporation of marine derived porous cuttlefish bone hydroxyapatite. *Materials (Basel)*.

[22] Papadogiannis Y., Helvatjoglou-Antoniadi M., Lakes R. C., Sapountjis M. (1991). The creep behavior of glass-ionomer restorative materials. *Dental Materials*.

[23] el Hejazi A. A., Watts D. C. (1999). Creep and visco-elastic recovery of cured and secondary-cured composites and resin-modified glass-ionomers. *Dental Materials*.

[24] Magni E., Ferrari M., Hickel R., Ilie N. (2010). Evaluation of the mechanical properties of dental adhesives and glass-ionomer cements. *Clinical Oral Investigations*.

[25] Coutinho E., Cardoso M. V., De Munck J. (2009). Bonding effectiveness and interfacial characterization of a nano-filled resin- modified glass-ionomer. *Dental Materials*.

[26] Peez R., Frank S. (2006). The physical-mechanical performance of the new Ketac™ Molar Easymix compared to commercially available glass ionomer restoratives. *Journal of Dentistry*.

[27] Magne P., Silva S., Andrada M., Maia H. (2016). Fatigue resistance and crack propensity of novel “super-closed” sandwich composite resin restorations in large MOD defects. *International Journal of Esthetic Dentistry*.

[28] Sulaiman T. A., Abdulmajeed A. A., Altitinchi A., Ahmed S. N., Donovan T. E. (2018). Effect of resin-modified glass ionomer cement dispensing/mixing methods on mechanical properties. *Operative Dentistry*.

[29] Oliveira G. L., Carvalho C. N., Carvalho E. M., Bauer J., Leal A. M. A. (2019). The influence of mixing methods on the compressive strength and fluoride release of conventional and resin-modified glass ionomer cements. *International Journal of Dentistry*.

[30] Al-Taee L., Deb S., Banerjee A. (2020). An in vitro assessment of the physical properties of manually- mixed and encapsulated glass-ionomer cements. *BDJ Open*.

[31] Santos M. J., Ari N., Steele S., Costella J., Banting D. (2014). Retention of tooth-colored restorations in non-carious cervical lesions—a systematic review. *Clinical Oral Investigation*.

[32] Donly K. J., Segura A., Kanellis M., Erickson R. L. (1999). Clinical performance and caries inhibition of resin-modified glass ionomer cement and amalgam restorations. *Journal of the American Dental Association*.

[33] Espelid I., Tveit A. B., Tornes K. H., Alvheim H. (1999). Clinical behaviour of glass ionomer restorations in primary teeth. *Journal of Dentistry*.

[34] Dülgergil C. T., Soyman M., Civelek A. (2005). Atraumatic restorative treatment with resin-modified glass ionomer material: short-term results of a pilot study. *Medical Principles and Practice*.

[35] Fagundes T. C., Barata T. J., Bresciani E. (2014). Seven-year clinical performance of resin composite versus resin-modified glass ionomer restorations in noncarious cervical lesions. *Operative Dentistry*.

[36] ISO 9917-2 (2017). *Dentistry-water-based cements-part 2: resin-modified cements*.

[37] Kitasako Y., Burrow M. F., Nikaido T., Tagami J. (2000). The influence of storage solution on dentin bond durability of resin cement. *Dental Materials*.

[38] Krämer N., Schmidt M., Lücker S., Domann E., Frankenberger R. (2018). Glass ionomer cement inhibits secondary caries in an in vitro biofilm model. *Clinical Oral Investigations*.

[39] Haghi H. V., Peeri-Dogaheh H., Fazlalizadeh S., Abazari M., Mohammadhosseini R. (2021). Effect of Streptococcus mutans on the flexural strength of resin-based restorative materials. *Dental Research Journal*.

[40] Hoshika S., Ting S., Ahmed Z. (2021). Effect of conditioning and 1 year aging on the bond strength and interfacial morphology of glass-ionomer cement bonded to dentin. *Dental Materials*.

[41] Vichitgomen J., Srisawasdi S. (2021). Deep margin elevation with resin composite and resin-modified glass-ionomer on marginal sealing of CAD-CAM ceramic inlays: an in vitro study. *American Journal of Dentistry*.

[42] ISO 5833 (2002). *Implats for surgery–acrylic resin cements*.

[43] Baker L. M., Freiman S. W. (1979). Short bar specimen for KIc measurements. *Fracture Mechanics Applied to Brittle Materials*.

[44] Perdigão J., Dutra-Corrêa M., Saraceni S. H., Ciaramicoli M. T., Kiyan V. H. (2012). Randomized clinical trial of two resin-modified glass ionomer materials: 1-year results. *Operative Dentistry*.

[45] Falsafi A., Mitra S. B., Oxman J. D., Ton T. T., Bui H. T. (2014). Mechanisms of setting reactions and interfacial behavior of a nano-filled resin-modified glass ionomer. *Dental Materials*.

[46] Mitchell C. A., Douglas W. H., Cheng Y. S. (1999). Fracture toughness of conventional, resin-modified glass-ionomer and composite luting cements. *Dental Materials*.

[47] Croll T. P., Berg J. H. (2007). Resin-modified glass-ionomer restoration of primary molars with proximating class II caries lesions. *Compendium if Continuing Education in Dentistry*.

[48] Killian C. M., Croll T. P. (2010). Nano-ionomer tooth repair in pediatric dentistry. *Pediatric Dentistry*.

[49] Ilie N., Hickel R., Valceanu A. S., Huth K. C. (2012). Fracture toughness of dental restorative materials. *Clinical Oral Investigations*.

[50] Kovarik R. E., Fairhurst C. W. (1993). Effect of Griffith precracks on measurement of composite fracture toughness. *Dental Materials*.

[51] Croll T. P., Bar-Zion Y., Segura A., Donly K. J. (2001). Clinical performance of resin-modified glass ionomer cement restorations in primary teeth: a retrospective evaluation. *Journal of the American Dental Association (1939)*.

[52] Toh S. L., Messer L. B. (2007). Evidence-based assessment of tooth-colored restorations in proximal lesions of primary molars. *Pediatric Dentistry*.

[53] Ausiello P., Ciaramella S., Di Rienzo A., Lanzotti A., Ventre M., Watts D. C. (2019). Adhesive class I restorations in sound molar teeth incorporating combined resin-composite and glass ionomer materials: CAD-FE modeling and analysis. *Dental Materials*.

[54] Mitsuhashi A., Hanaoka K., Teranaka T. (2003). Fracture toughness of resin-modified glass ionomer restorative materials: effect of powder/liquid ratio and powder particle size reduction on fracture toughness. *Dental Materials*.

[55] Dowling A. H., Fleming G. J. (2008). Is encapsulation of posterior glass-ionomer restoratives the solution to clinically induced variability introduced on mixing?. *Dental Materials*.

[56] Li Y., Lin H., Zheng G., Zhang X., Xu Y. (2015). A comparison study on the flexural strength and compressive strength of four resin-modified luting glass ionomer cements. *Bio-medical Materials and Engineering*.

[57] Moreau J. L., Xu H. H. (2010). Fluoride releasing restorative materials: effects of pH on mechanical properties and ion release. *Dental Materials*.

[58] Gjorgievska E., Van Tendeloo G., Nicholson J. W., Coleman N. J., Slipper I. J., Booth S. (2015). The incorporation of nanoparticles into conventional glass-ionomer dental restorative cements. *Microscopy and Microanalysis*.

[59] Gjorgievska E., Nicholson J. W., Gabrić D., Guclu Z. A., Miletić I., Coleman N. J. (2020). Assessment of the impact of the addition of nanoparticles on the properties of glass-ionomer cements. *Materials (Basel)*.

[60] Nicholson J. W., Sidhu S. K., Czarnecka B. (2020). Enhancing the mechanical properties of glass-ionomer dental cements: a review. *Materials (Basel)*.

[61] Davis D. M., Waters N. E. (1987). An investigation into the fracture behavior of a particulate-filled bis-GMA resin. *Journal of Dental Research*.

[62] Ferracane J. L., Antonio R. C., Matsumoto H. (1987). Variables affecting the fracture toughness of dental composites. *Journal of Dental Research*.

[63] Kim K. H., Park J. H., Imai Y., Kishi T. (1994). Microfracture mechanisms of dental resin composites containing spherically-shaped filler particles. *Journal of Dental Research*.

[64] Alvanforoush N., Wong R., Burrow M., Palamara J. (2019). Fracture toughness of glass ionomers measured with two different methods. *Journal of the Mechanical Behavior of Biomedical Materials*.

[65] Garoushi S., Vallittu P., Lassila L. (2017). Hollow glass fibers in reinforcing glass ionomer cements. *Dental Materials*.

[66] Mederos M., Cuevas-Suarez C. E., Sanchez W. (2021). Effect of the incorporation of hydroxyapatite on the diametral tensile strength of conventional and hybrid glass ionomer cements. *Odontology*.

[67] Beriat N. C., Nalbant D. (2009). Water absorption and HEMA release of resin-modified glass-ionomers. *European Journal Of Dentistry*.

[68] Mitra S. B. (1991). Adhesion to dentin and physical properties of a light-cured glass-ionomer liner/base. *Journal of Dental Research*.

[69] Zankuli M. A., Silikas N., Devlin H. (2015). The effect of cyclic loading on the compressive strength of core build-up materials. *Journal of Prosthodontics*.

[70] Aratani M., Pereira A. C., Correr-Sobrinho L., Sinhoreti M. A., Consani S. (2005). Compressive strength of resin-modified glass ionomer restorative material: effect of P/L ratio and storage time. *Journal of Applied Oral Science*.

