# Supplementary material for: Evaluation of the Mechanical Properties of Three Resin-Modified Glass-Ionomer Materials
Source: Biomed Res Int. 2022 Aug 2;2022:4690656. doi: 10.1155/2022/4690656 (PMC9363206; doi:10.1155/2022/4690656)
Supplement: Supplementary Materials — The statistical analysis (raw data) is available for the diametral tensile strength, flexural strength, and fracture toughness of the RMGI materials tested. [file 4690656.f1.zip › Diametral Tensile Strength of RMGI.pdf]

Ketac 7h	Riva 7h	Fuji II 7h	Ketac 32h	Riva 32h	Fuji II 32h
10.80	14.04	20.78	8.68	18.45	14.50
8.64	9.98	24.25	8.59	17.79	23.29
9.22	13.65	17.32	17.99	7.93	18.91
10.97	13.04	16.06	15.84	10.82	24.78
14.24	10.63	24.11	9.56	11.23	22.83
12.58	12.62	24.13	6.81	11.91	23.47
10.76	12.05	16.55	22.42	13.82	22.42
6.81	19.88	15.09	15.30	12.79	19.91
9.93	14.03	25.89	15.18	15.19	17.69
13.85	13.03	25.11	16.98	9.25	17.39

Table Analyzed	Data 1				
ANOVA summary					
F	13.83				
P value	< 0.0001				
P value summary	****				
Are differences among means statistically significant? (P < 0.05)	Yes				
R square	0.5615				
Brown-Forsythe test					
F (DFn, DFd)	2.028 (5, 54)				
P value	0.0893				
P value summary	ns				
Significantly different standard deviations? (P < 0.05)	No				
Bartlett's test					
Bartlett's statistic (corrected)	7.009				
P value	0.2200				
P value summary	ns				
Significantly different standard deviations? (P < 0.05)	No				
ANOVA table	SS	DF	MS	F (DFn, DFd)	P value
Treatment (between columns)	914.7	5	182.9	F (5, 54) = 13.83	P < 0.0001
Residual (within columns)	714.4	54	13.23		
Total	1629	59			
Data summary					
Number of treatments (columns)	6				
Number of values (total)	60				

Number of families	1			
Number of comparisons per family	15			
Alpha	0.05			
Tukey's multiple comparisons test	Mean Diff.	95% CI of diff.	Significant?	Summary
Ketac 7h vs. Riva 7h	-2.515	-7.321 to 2.291	No	ns
Ketac 7h vs. Fuji II 7h	-10.15	-14.95 to -5.343	Yes	****
Ketac 7h vs. Ketac 32h	-2.955	-7.761 to 1.851	No	ns
Ketac 7h vs. Riva 32h	-2.138	-6.944 to 2.668	No	ns
Ketac 7h vs. Fuji II 32h	-9.739	-14.54 to -4.933	Yes	****
Riva 7h vs. Fuji II 7h	-7.634	-12.44 to -2.828	Yes	***
Riva 7h vs. Ketac 32h	-0.4400	-5.246 to 4.366	No	ns
Riva 7h vs. Riva 32h	0.3770	-4.429 to 5.183	No	ns
Riva 7h vs. Fuji II 32h	-7.224	-12.03 to -2.418	Yes	***
Fuji II 7h vs. Ketac 32h	7.194	2.388 to 12.00	Yes	***
Fuji II 7h vs. Riva 32h	8.011	3.205 to 12.82	Yes	***
Fuji II 7h vs. Fuji II 32h	0.4100	-4.396 to 5.216	No	ns
Ketac 32h vs. Riva 32h	0.8170	-3.989 to 5.623	No	ns
Ketac 32h vs. Fuji II 32h	-6.784	-11.59 to -1.978	Yes	**
Riva 32h vs. Fuji II 32h	-7.601	-12.41 to -2.795	Yes	***

Test details	Mean 1	Mean 2	Mean Diff.	SE of diff.	n1	n2	q	DF
Ketac 7h vs. Riva 7h	10.78	13.30	-2.515	1.627	10	10	2.187	54
Ketac 7h vs. Fuji II 7h	10.78	20.93	-10.15	1.627	10	10	8.824	54
Ketac 7h vs. Ketac 32h	10.78	13.74	-2.955	1.627	10	10	2.569	54
Ketac 7h vs. Riva 32h	10.78	12.92	-2.138	1.627	10	10	1.859	54
Ketac 7h vs. Fuji II 32h	10.78	20.52	-9.739	1.627	10	10	8.468	54
Riva 7h vs. Fuji II 7h	13.30	20.93	-7.634	1.627	10	10	6.637	54
Riva 7h vs. Ketac 32h	13.30	13.74	-0.4400	1.627	10	10	0.3826	54
Riva 7h vs. Riva 32h	13.30	12.92	0.3770	1.627	10	10	0.3278	54
Riva 7h vs. Fuji II 32h	13.30	20.52	-7.224	1.627	10	10	6.281	54
Fuji II 7h vs. Ketac 32h	20.93	13.74	7.194	1.627	10	10	6.255	54
Fuji II 7h vs. Riva 32h	20.93	12.92	8.011	1.627	10	10	6.965	54
Fuji II 7h vs. Fuji II 32h	20.93	20.52	0.4100	1.627	10	10	0.3565	54
Ketac 32h vs. Riva 32h	13.74	12.92	0.8170	1.627	10	10	0.7103	54
Ketac 32h vs. Fuji II 32h	13.74	20.52	-6.784	1.627	10	10	5.898	54
Riva 32h vs. Fuji II 32h	12.92	20.52	-7.601	1.627	10	10	6.609	54

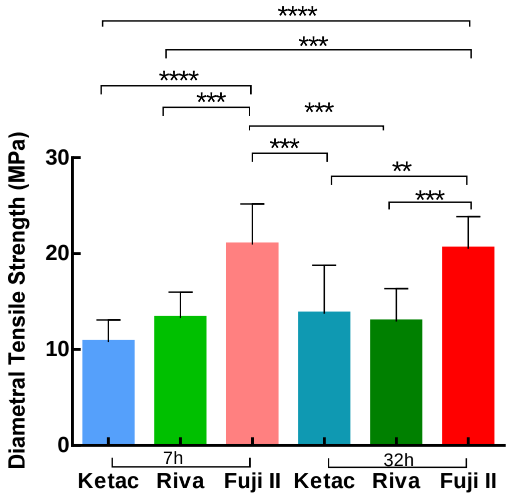

Diametral Tensile Strength (MPa)
0
10
20
30
****
***
****
***
***
***
**
***
7h
32h
Ketac
Riva
Fuji II
Ketac
Riva
Fuji II
