# Supplementary material for: Evaluation of the Mechanical Properties of Three Resin-Modified Glass-Ionomer Materials
Source: Biomed Res Int. 2022 Aug 2;2022:4690656. doi: 10.1155/2022/4690656 (PMC9363206; doi:10.1155/2022/4690656)

Ketac	Riva	Fuji II
14.84	19.78	47.12
13.48	15.33	38.81
11.83	17.19	39.40
13.14	15.35	33.69
15.09	14.23	44.07
13.79	14.80	28.82
16.12	17.69	36.29
12.21	14.38	35.29
14.29	14.55	39.05
15.62	13.35	33.49
14.04	15.67	37.60
1.42	1.96	5.33

Table Analyzed	Data 1				
ANOVA summary					
F	37.51				
P value	< 0.0001				
P value summary	****				
Are differences among means statistically significant? (P < 0.05)	Yes				
R square	0.6945				
Brown-Forsythe test					
F (DFn, DFd)	1.889 (2, 33)				
P value	0.1672				
P value summary	ns				
Significantly different standard deviations? (P < 0.05)	No				
Bartlett's test					
Bartlett's statistic (corrected)	13.44				
P value	0.0012				
P value summary	**				
Significantly different standard deviations? (P < 0.05)	Yes				
ANOVA table	SS	DF	MS	F (DFn, DFd)	P value
Treatment (between columns)	3595	2	1798	F (2, 33) = 37.51	P < 0.0001
Residual (within columns)	1581	33	47.92		
Total	5176	35			

Number of families	1
Number of comparisons per family	3
Alpha	0.05

Tukey's multiple comparisons test	Mean Diff.	95% CI of diff.	Significant?	Summary
Ketac vs. Riva	-1.534	-8.468 to 5.400	No	ns
Ketac vs. Fuji II	-21.92	-28.86 to -14.99	Yes	****
Riva vs. Fuji II	-20.39	-27.32 to -13.46	Yes	****

Test details	Mean 1	Mean 2	Mean Diff.	SE of diff.	n1	n2	q	DF
Ketac vs. Riva	12.99	14.52	-1.534	2.826	12	12	0.7678	33
Ketac vs. Fuji II	12.99	34.91	-21.92	2.826	12	12	10.97	33
Riva vs. Fuji II	14.52	34.91	-20.39	2.826	12	12	10.20	33

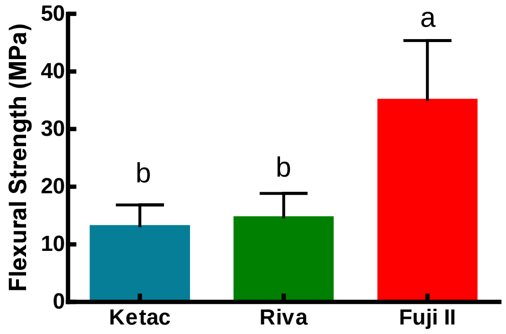

Supplement: Supplementary Materials — The statistical analysis (raw data) is available for the diametral tensile strength, flexural strength, and fracture toughness of the RMGI materials tested. [file 4690656.f1.zip › Flexural Strength of RMGI.pdf]
