# Supplementary material for: Evaluation of the Mechanical Properties of Three Resin-Modified Glass-Ionomer Materials
Source: Biomed Res Int. 2022 Aug 2;2022:4690656. doi: 10.1155/2022/4690656 (PMC9363206; doi:10.1155/2022/4690656)

Ketac	Riva	Fuji II
0.18	0.17	0.43
0.15	0.19	0.22
0.18	0.24	0.21
0.13	0.16	0.27
0.16	0.21	0.23
0.18	0.18	0.38
0.10	0.22	0.25
0.12	0.16	0.29
0.17	0.21	0.22
0.11	0.24	0.21
0.15	***0.20***	***0.27***
0.03	***0.03***	***0.08***

Table Analyzed	Data 1				
ANOVA summary					
F	14.93				
P value	< 0.0001				
P value summary	****				
Are differences among means statistically significant? (P < 0.05)	Yes				
R square	0.5251				
Brown-Forsythe test					
F (DFn, DFd)	1.738 (2, 27)				
P value	0.1949				
P value summary	ns				
Significantly different standard deviations? (P < 0.05)	No				
Bartlett's test					
Bartlett's statistic (corrected)	10.19				
P value	0.0061				
P value summary	**				
Significantly different standard deviations? (P < 0.05)	Yes				
ANOVA table	SS	DF	MS	F (DFn, DFd)	P value
Treatment (between columns)	0.07653	2	0.03826	F (2, 27) = 14.93	P < 0.0001

Residual (within columns)	0.06921	27	0.002563
Total	0.1457	29	
Data summary			
Number of treatments (columns)	3		
Number of values (total)	30		

Number of families	1
Number of comparisons per family	3
Alpha	0.05

Tukey's multiple comparisons test	Mean Diff.	95% CI of diff.	Significant?	Summary
Ketac vs. Riva	-0.0500	-0.1061 to 0.006139	No	ns
Ketac vs. Fuji II	-0.1230	-0.1791 to -0.06686	Yes	****
Riva vs. Fuji II	-0.0730	-0.1291 to -0.01686	Yes	**

Test details	Mean 1	Mean 2	Mean Diff.	SE of diff.	n1	n2	q
Ketac vs. Riva	0.1480	0.1980	-0.0500	0.02264	10	10	3.123
Ketac vs. Fuji II	0.1480	0.2710	-0.1230	0.02264	10	10	7.683
Riva vs. Fuji II	0.1980	0.2710	-0.0730	0.02264	10	10	4.560

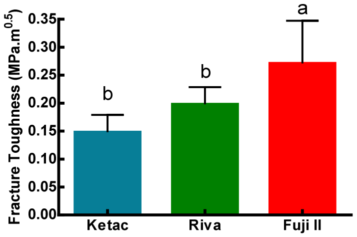

Supplement: Supplementary Materials — The statistical analysis (raw data) is available for the diametral tensile strength, flexural strength, and fracture toughness of the RMGI materials tested. [file 4690656.f1.zip › Fracture Toughness of RMGI.pdf]
